# How to diagnose iron deficiency in chronic disease: A review of current methods and potential marker for the outcome

**DOI:** 10.1186/s40001-022-00922-6

**Published:** 2023-01-09

**Authors:** Martina Rohr, Vincent Brandenburg, Hans-Peter Brunner-La Rocca

**Affiliations:** 1 Vifor Pharma Deutschland GmbH, Baierbrunner Straße 29, 81379 Munich, Germany; 2 Dept of Cardiology and Nephrology, Rhein-Maas Klinikum Würselen, Mauerfeldchen 25, 52146 Würselen, Germany; 3 Cardiovascular Research Institute Maastricht (CARIM), Maastricht, The Netherlands; 4 Department of Cardiology, MUMC+, Maastricht University Medical Centre, P. Debyelaan 25, Main Building, 3rd Floor, room 3.B2.022, 6229 HX Maastricht, The Netherlands; 5 PO Box 5800, 6202 AZ Maastricht, The Netherlands

## Abstract

Iron deficiency (ID) is the most common nutritional disorder worldwide. It is often observed in patients with chronic diseases, such as heart failure (HF), chronic kidney disease (CKD), inflammatory bowel disease (IBD) and cancer. ID is associated with poor clinical outcome, including poor performance, reduced quality of life, as well as increased hospitalization and mortality. The aim of this review is to provide an overview about the role of ID in chronic diseases (HF, CKD, IBD, cancer) regarding their current definitions and clinical relevance; diagnostic accuracy of iron parameters in chronic inflammatory conditions and its potential as prognostic markers. Due to different definitions and guideline recommendations of ID, various laboratory parameters for ID diagnostic exist and there is no general consensus about the definition of ID and its treatment. Still, a general trend can be observed across all investigated indications of this review (HF, CKD, IBD, cancer) that serum ferritin and transferrin saturation (TSAT) are the two parameters mentioned most often and emphasized in all guidelines to define ID and guide treatment. The most commonly used threshold values for the diagnosis of ID are TSAT of < 20% and serum ferritin of < 100–300 µg/L. Noteworthy, both TSAT and particularly ferritin are frequently applied, but both may vary due to inflammatory conditions. Studies showed that TSAT is less affected by inflammatory processes and may therefore be more accurate and reliable than serum ferritin, particularly in conditions with elevated inflammatory state. A low iron status and particularly a low TSAT value was associated with a poor outcome in all investigated indications, with the strongest evidence in HF patients. Routine surveillance of iron status in these groups of patients with chronic conditions is advisable to detect ID early. Depending on the inflammatory state, TSAT < 20% may be the more accurate diagnostic marker of ID than ferritin. Moreover, TSAT may also be the more reliable estimate for the prognosis, particularly in HF.

## Introduction

Iron deficiency (ID) is the most common nutritional disorder in the world [1]. Multifactorial causes can lead to ID, of which blood loss, insufficient dietary intake and reduced iron absorption and metabolism due to inflammatory conditions are the most important ones [2, 3]. ID is associated with a worse clinical outcome such as poor performance, reduced quality of life, increased hospitalization and mortality [4, 5]. Moreover, ID may also aggravate underlying chronic diseases [4–6]; e.g., heart failure (HF) patients with ID have more symptoms and worse outcome [7, 8] or patients with chronic kidney disease (CKD) plus ID progress more often to end-stage kidney disease [9, 10]. ID is often overlooked, as the most common symptoms of ID (e.g., fatigue, headache, hair loss, sleeplessness) are unspecific and often difficult to distinguish from symptoms of the primary disease [4, 11]. Moreover, ID and the presence of anemia (iron-deficiency anemia) are not necessarily linked with each other, i.e., each can be present in the absent of the other [4]. Hence, screening for hemoglobin concentration and red blood cell indices is insufficient to adequately diagnose ID [11]. In addition, the definition of ID is not consistent [5]. Thus, a uniform diagnosis would be necessary for an accurate identification of ID, based on its specific pathophysiological role, independent of anemia [12]. The aim of this review is to provide an illustrative overview and comparison of the role of ID in different chronic diseases. Therefore, we defined three core themes of this review:

1. To evaluate the current definitions of ID and their clinical relevance;
2. To discuss the diagnostic accuracy of iron parameters in chronic inflammatory conditions;
3. To assess the role of iron parameters as prognostic markers.

### Pathophysiology of ID in chronic inflammation

In case of inflammatory conditions, the availability of iron is limited, this includes reduced bioavailability by down-regulated intestinal absorption as well as transfer from iron storages and utilization in the bone marrow [5]. The mechanism of reduced iron metabolism is complex and still not fully understood, but the peptide hepcidin plays a key role as master switch in the regulation of iron uptake and distribution in inflammatory conditions [13, 14]. Studies examined pathophysiologic processes of ID in inflammatory conditions, showing that ID is triggered by pro-inflammatory cytokines (IL-6, IL-1, TNF-α). Cytokine-activated signaling pathways lead to blunted responses to erythropoietin (EPO), apoptosis of erythroid progenitors and hepcidin-mediated malabsorption and reticuloendothelial system (RES) trapping of iron [13]. Hepcidin is an antimicrobial peptide hormone and is mainly expressed in the liver due to cytokine activation such as IL-6. Hepcidin regulates iron by binding to and inducing internalization of ferroportin, the only known cellular iron-exporting protein [13]. Ferroportin channels are present on the basolateral membrane of enterocytes, hepatocytes and macrophages, and iron transport is inhibited by an elevated hepcidin level [13, 14]. Hence, hepcidin can be described as a central gate-keeper, which is triggered by inflammatory conditions and contributes to ID, especially in chronic diseases [4, 5, 13].

## Definition of iron deficiency

Understanding the pathophysiology of ID is also essential to properly define the iron status and for subsequent diagnosis. Alterations in iron availability can be described in terms of ‘absolute’ or ‘functional’ ID [15]. The reduction of stored iron in the monocyte-macrophage system, including bone marrow, liver and spleen is referred to as absolute ID. This is characterized by a low level of stored iron resulting in low serum ferritin and a reduced saturation of the iron transport protein transferrin (i.e., low level of transferrin saturation—TSAT). Functional ID is described by normal iron stores, but iron cannot be released sufficiently due to internalized ferroportin channels, caused by hepcidin [11, 16]. This results in normal ferritin levels, but iron is not sufficiently available for the system [13, 16]. As ferritin is an acute phase protein, it may be even increased, whereas TSAT may be less affected by inflammatory effects. The low availability of iron results in a low TSAT level [5]. Inflammatory processes are not absolute static as the extent and effects of chronic inflammation depend on several internal and external pathogenic factors, resulting in significant changes in inflammation status and disease activity, resulting in changes in iron availability [4, 5, 13]. Thus, the definition of ID should reflect the body’s needs of iron in the context of its iron availability. Importantly, ID needs to be clearly distinguished from ID anemia as ID and ID anemia have different physiological characteristics with their individual symptomatic, diagnostic and treatment profiles [4].

### Diagnosis of iron deficiency

The gold standard to determine ID is to determine iron content of the bone marrow. However, this is not feasible in routine clinical practice [17]. Therefore, different non-invasive biochemical markers are widely used to determine ID.

#### Types of biochemical markers

##### Serum iron

In case of ID, serum iron falls below the normal range of 40–165 μg/L [3]. Assessing iron status solely upon serum iron measurement is limited due to rapid fluctuations of serum iron depending, e.g., on nutritional intake and circadian rhythm [11]. It is reduced by inflammation [11, 14]. The variability of serum iron concentrations, which are necessary for determination of TSAT, should be included for interpretation of TSAT results [5].

##### Serum transferrin

Transferrin is a plasma glycoprotein mainly synthesized in the liver. The two binding sites for Fe^3+^ ions enable the transport of iron in the body to target cells for absorption by transferrin receptors. The binding affinity to transferrin is dependent on the pH value. Thus, iron-bound transferrin circulates with a high affinity in the healthy plasma and releases iron in endosomes with acidic pH [14, 18]. In case of ID and hypoxia, transferrin synthesis rate increases. Steroid hormones (e.g., estrogens) also stimulate transferrin synthesis, but it is inhibited by lack of nutrients. In addition, inflammatory conditions have a negative impact on synthesis of transferrin, but data are not consistent [14]. Normal serum transferrin conc. is 200–400 mg/dL [11]. For the diagnosis of ID, transferrin is mainly used for the calculation of transferrin saturation [14].

##### Transferrin saturation (TSAT)

TSAT provides information about iron availability. It is calculated from serum iron concentration and the total iron binding capacity (TIBC) and is expressed in %. The TIBC reflects the blood’s binding capacity of iron and is very closely correlated with transferrin [11, 18, 19]. Under normal conditions, 20–45% of the TICB is loaded with iron (= TSAT) [18].

$$\text {Total iron binding capacity (TIBC)\,=\,serum Transferrin x conversion factor }$$

converting factor (25.1 for TIBC (μmol/L) or 1.4 for TIBC (μg/L)) [18]

$$\mathrm{TSAT }\left(\mathrm{\%}\right)= \frac{\text{Serum iron }}{\text{TIBC }(\text{serum transferrin x conversion factor})}\mathrm{x }100$$

In ID, TSAT usually falls below 20% [18]. TSAT as a reliable diagnostic maker for ID is often used in clinical practice and recommended in several guidelines and studies [11, 20–23] with high sensitivity and specificity [24]. Noteworthy, the value of TSAT may fluctuate as both serum iron and transferrin are influenced by various factors as addressed above [11, 14]. Due to the influence of the circadian rhythm and dietary intake of iron, TSAT should always be measured in the morning under fasting conditions [5]. The impact of inflammation seems to be negligible, because studies show that TSAT is fluctuating less than serum ferritin in inflammatory conditions [2, 5, 24]. Therefore, TSAT can be used to determine ID with chronic diseases [2, 4].

##### Serum ferritin

Ferritin is a marker to evaluate the iron stores. Decreased serum ferritin with a threshold of < 30 μg/L in otherwise healthy individuals or < 100 μg/L in chronic conditions indicates an inadequate level of iron reservoir and thereby absolute ID [5]. However, the thresholds of serum ferritin concentrations may vary depending on age. Ferritin values are higher at birth, decrease in childhood and increase again in adulthood [17]. Low ferritin levels are also observed in elderly patients due to iron resorption disorders or lower food intake [5].

In inflammatory processes, ferritin acts as an acute phase protein leading to increased values [5]. Other factors, such as liver disease/damage, multiple malignancies, infections, renal failure, muscular cytolysis, decompensated diabetes and hyperthyroidism and certain metabolic syndromes can also cause increased ferritin levels [14, 25]. An increased ferritin value occurs after iron administration or blood transfusion, which may influence the accuracy of the ID diagnosis [14, 26]. Falsely low ferritin values independent of iron depletion are reported in hypothyroidism or vitamin C deficiency [17]. In addition, a high variability between the laboratory methods for serum ferritin measurement was found [5, 25]. Therefore, ferritin alone is not suitable for the diagnosis of ID, especially in inflammatory conditions. If acute or chronic inflammation is suspected, the CRP (C-reactive protein) value should be determined as well [5].

##### Soluble transferrin receptor (sTfR)

The sTfR is a protein dimer that results from proteolytic cleavage of the extracellular transferrin receptor on cell surfaces. The serum concentration is directly proportional to the erythropoietic rate and inversely proportional to tissue iron availability [27]. The normal range of sTfR is 0.8–2.2 mg/L [3]. In iron-deficient patients and generally in every expansion of erythropoiesis, increased sTfR levels (> 1.8 m/L (Dade-Behring test); > 4.4 mg/L for women and > 5.0 mg/L for men (Roche test)) are observed [11]. Therefore, increased levels can also be shown in patients with hemolysis or after administration of erythropoiesis-stimulating agents (ESAs). Reduced sTfR levels were found in conditions of hypoproliferative erythropoiesis (e.g., aplastic anemia or renal anemia) [3, 11]. The main advantage of sTfR is the reflection of the entire erythropoiesis, which is useful for a differential diagnosis in iron-deficient erythropoiesis. A disadvantage of this parameter might be its inaccuracy in case of functional ID, where false normal sTfR values are measured due to inhibited erythropoiesis [11]. Regarding its clinical use, the calculation of the sTfR–ferritin index is recommended [3].

##### sTfR–ferritin index

The ratio of sTfR (in mg/L) and the log of ferritin (in μg/L), is called sTfR–ferritin index (sTfR/log-ferritin). This index is convenient for the diagnosis of functional ID in inflammatory conditions, and also for anemia of chronic diseases (ACD). The sTfR reflects the erythropoiesis and increases with ID, whereas ferritin describes decreased iron stores in case of ID, and is normal or increased in ACD [11]. The threshold of sTfR–ferritin index depends on inflammatory conditions and individual assay methods. For absolute ID, the threshold of sTfR–ferritin index is > 1.5 (Dade-Behring test) or > 3.2 (Roche test), whereas for functional ID (CRP > 5 mg/L) and ACD (reduced hemoglobin (Hb) and CRP > 5 mg/L) > 0.8 (Dade-Behring test) or > 2.0 (Roche test) [11, 28]. In general, the sTfR–ferritin index is highly specific [29]. Several factors limit the use of this parameter in clinical practice such as high costs, availability of heterogeneous assays and different sTfR norms [3, 15].

#### Guideline recommendations for ID diagnostics

A total of 12 guidelines in chronic disease (HF, CKD, IBD, cancer) [11, 20, 21, 23, 30–37] and 3 guidelines in general population [38–40] were identified that deal with the diagnosis of ID (Table 1). All guidelines recommended the screening for ID, but their definitions of ID vary significantly. However, all guidelines recommend to measure ferritin concentrations and nearly three-quarters (11 of 15) propose TSAT as an alternative or complementary parameter for ID diagnosis, especially in the presence of inflammation and/or for functional ID in chronic disease.

**Table 1:** Guideline definition of ID in general population and chronic disease; summary of current ID laboratory measurements

Professional association	Origin	Year	Condition	Threshold values for ID diagnostic		
				Serum ferritin (μg/L)	TSAT (%)	Additional biomarkers
*General population*						
WHO [38]	International	2020	ID in		–	sTfR#, CRP**
			˗ Infants and children < 5 years	< 12		
			* with* infection/inflammation	< 30		
			˗ Children > 5 years, adolescents, adults, older person (60 + years)	< 15		
			* with* infection/inflammation	< 70		
			˗ Pregnant women (*only for first trimester*)	< 15		
AAFP [39]	American	2013	IDA	< 30	–	low serum iron^#^, low TSAT^#^, high TIBC^#^
			*with* infection/ inflammation	< 50		
BCG [40]	British	2019	ID in children	< 12	additional < 20	serum iron^#^, TIBC^#^, TSAT
			- Possible ID	12–20		
			ID in adults	< 15		
			- Probable ID	15–30		
			- Unlikely ID	> 30		
			* or* with chronic inflammatory disease	> 70–100		
			* or* in elderly patients	> 50		
*Chronic heart failure (HF)*						
ACCF/AHA [30]	American	2017	AID	< 100	–	–
			FID	or 100–300	and < 20	
ESC [23]	European	2021	AID	< 100	and < 20	sTfR^#^
			FID	or 100–299	and < 20	
*Chronic kidney disease (CKD)*						
KIDIGO [31]	International	2012	IDA only	–	–	–
			ND/HD-CKD + IDA	≤ 500	and ≤ 30	
			Pediatric CKD + IDA	≤ 100	and ≤ 20	
			Severe ID	≤ 30	–	
KDOQI [32]	American	2013	IDA only	–	–	–
ERBP [33]	European	2013	ID/IDA (AID)	< 100	and < 20	–
UK NICE [34]	British	2021	IDA only	< 100*	and < 20*	Red blood cell markers: %HRC > 6%, CHr < 29 pg
JSDT [35] (2015 version)	Japanese	2017	HD-CKD + ID	≤ 100	≤ 20	–
*Inflammatory bowel disease (IBD)*						
ECCO [21]	European	2015	ID/IDA (quiescent disease, without inflammation)	< 30	–	Tests to distinguish between IDA and ACD: sTfR, sTfR–ferritin index
			AID and ACD	30–100	and/or < 20	
			FID and ACD	> 100	and < 20	
BSG [36]	British	2021	ID/IDA (AID)	< 15	–	sTfR^#^ (in healthy subjects), sTfR–ferritin index^#^, CHr^#^, HYPO%^#^ (in chronic disease and/or raised ESR^#^, CRP)
			FID	≥ 100	< 20	
*Cancer*						
NCCN [37]	America	2021	ID/IDA (AID)	< 30	and < 20	–
			FID (often when receiving ESAs)	30–500	and < 50	
			Possible FID	> 500–800	and < 50	
ESMO [20]	European	2018	ID/IDA (AID)	< 30–100	and < 20	AID: sTfR^#^, ZPP^#^ AID and FID: %HYPO > 5%, CHr < 28 pg, CRP**
			FID	> 100	and < 20	
DGHO [11]	Germany	2022	ID/IDA (AID)	< 30–100	< 20	sTfR > 0.76–1.76 mg/L (*Dade Behring Test*), sTfR–ferritin index > 0.2–3.7 men, > 0.6–3.8 women (*Roche Test*), ZPP > 80 µmol/mol Häm, HYPO% > 10%, CHr < 26 pg, CRP**
			FID	100–800	and < 20	

*%HRC* percentage of hypochromic red cells, *AAFP* American Academy of Family Physicians, *ACCF* American College of Cardiology Foundation, *ACD* Anemia of chronic disease, *AHA* American Heart Association, *AID* absolute iron deficiency, *BCG* British Columbia Guidance, *BSG* British Society of Gastroenterology, *CHr* reticulocyte hemoglobin content, *CRP* C-reactive protein, *DGHO* Deutsche Gesellschaft für Hämatologie und Onkologie, *ECCO* European Crohn’s and Colitis Organisation, *ERBP* European Renal Best Practice, *ESA* Erythropoiesis-Stimulating Agents, *ESC* European Society of Cardiology, *ESMO* European Society for Medical Oncology, *ESR* erythrocyte sedimentation rate, FID functional iron deficiency, *HYPO%* hypochromic erythrocytes, *ID* iron deficiency, *IDA* iron deficiency anemia, *JSDT* Japanese Society for Dialysis Therapy, *KDIGO* Kidney Disease Improving Global Outcomes, *KDOQI* Kidney Disease Outcomes Quality Initiative, *NCCN* National Comprehensive Cancer Network, *NICE* National Clinical Guideline Centre, *sTfR* soluble transferrin receptor, *TSAT* transferrin saturation, *WHO* World Health Organization, *ZPP* zinc protoporphyrin

^*^ Second line, not recommended as stand-alone measurement

^**^ Inflammatory condition [CRP < 5 mg/L]

^#^ no cut-off values indicated

## Iron deficiency diagnosis and outcome in chronic diseases

### Iron deficiency in heart failure (HF)

A large number of patients with HF suffer from ID [8]. The etiology of ID in HF is multifactorial. It often arises from reduced iron intake, impaired intestinal absorption, gastrointestinal bleeding (e.g., by concomitant use of aspirin), uraemia (e.g., CKD), venepuncture, chronic low-grade inflammation or inhibition of EPO synthesis (CKD, possible due to use of angiotensin-converting inhibitors (ACE-I) or angiotensin receptor blockers (ARB)) [41, 42]. Different mechanism of EPO inhibition by ACE-I and ARB have been described [42, 43]. One is the inhibition of circulating angiotensin II levels resulting in lower levels of erythroid progenitor cells [44]. Other studies have demonstrated that levels of insulin-like growth factor 1 are reduced, which is associated with erythroid stimulation [45]. Another is the increasing level of the erythropoiesis inhibitor N-acetyl-seryl-aspartyl-lysine-proline by ACE-I use, leading to a decrease in erythropoiesis [46].

The prevalence of ID ranges from 33–74%, depending on the clinical study characteristics and the applied definition of ID [47]. Within an international pooled cohort of 1506 patients with chronic HF, an ID prevalence of 50% was found, defined by serum ferritin < 100 μg/L or serum ferritin 100–299 μg/L with TSAT < 20% [8], a definition later also used in an expert consensus document [48]. The recommendations are emphasized by the significant clinical impact of ID in patients with HF (Table 2), regardless of ejection fraction or disease severity [7, 8]. ID as a medical condition uncoupled from ID anemia was demonstrated in many trials with HF patients. It was shown that the presence of ID affects outcome [7, 49], irrespective of anemia [8, 50]. Patients with HF and ID were associated with poor health status, higher morbidity and mortality [51–56]. In some studies, negative outcomes were directly associated with low TSAT levels regardless of ferritin [24, 55]. For example, Ambrosy et al. (2020), showed a prevalence of TSAT < 20% in approx. 50% of the cases, which was associated with higher rates of morbidity and mortality [55]. A persistence of ID with TSAT < 20% in HF patients both at baseline and at 6 months was strongly associated with higher mortality compared with never having ID, superior to ID based on serum ferritin levels [57]. Thus, TSAT may be used as a marker for prognosis in HF patients [57, 58]. As to whether severity of ID is associated with worse outcome is not well investigated. Still, an association between sTfR and health-related quality of life has been found [59].

In conclusion, the best clinical outcomes in patients with HF were observed in patients with healthy iron status (TSAT > 20% and ferritin > 100 µg/L). ID was generally diagnosed with ferritin < 100 µg/L or 100–300 µg/L plus TSAT < 20%. Other iron parameters like the sTfR were rarely used or recommended for ID diagnosis in HF patients. The benefit of treating ID is an independent therapeutic target in HF [4]. Clinical evidence showed that there is a positive effect on management of ID with iv iron (FCM, ferric carboxymaltose) in outcomes: Compared to placebo, the administration of FCM improved NYHA class [60], prolonged 6-min walk test (6MWT) distance [61] and also decreased rates of recurrent cardiovascular (CV) hospitalizations and mortality [62] in HF patients with ID. These findings are supported in the recent AFFIRM-HF study, whereby treatment with FCM significantly reduced the risk of subsequent HF hospitalizations or CV death in iron-deficient HF patients compared to placebo [63]. In a secondary analysis of AFFIRM-HF, patients assigned to FCM had significantly increased health-related QoL [64].

**Table 2:** Iron deficiency-associated outcome in patients with heart failure (HF)

Study	Study population	ID definition / iron status	ID / iron status-associated outcome
Jankowska et al. (2011) [49]	ID and stable systolic CHF	Serum ferritin < 100 μg/L *or* Serum ferritin 100–300 μg/L + TSAT < 20%	• Exercise capacity: reduced peak oxygen consumption VO_2_ and increased ventilatory response to exercise VE-VCO_2_ slope
Klip et al. (2013) [8]	ID and CHF	Serum ferritin < 100 μg/L *or* Serum ferritin 100–299 μg/L + TSAT < 20%	• Higher NYHA class • Higher NT-proBNP levels • Lower mean corpuscular volume levels • Higher risk of morality
Comín-Colet et al. (2013) [50]	ID and/or IDA and CHF	Serum ferritin < 100 µg/L *or* Serum ferritin < 800 µg/L + TSAT < 20% *or* sTfR ≥ 1.62 mg/L	• Worse QoL (assessed with MLHFQ)
Núñez et al. (2016) [56]	ID and AHF	**AID**: serum ferritin < 100 μg/L *or* **FID**: serum ferritin 100–299 μg/L + TSAT < 20%	• Increased risk of early rehospitalization (only for AID)
Moliner et al. (2017) [52]	ID and CHF	Serum ferritin < 100 µg/L *and/or* TSAT < 20%	• Higher NT-proBNP levels* • Worse QoL* • Higher risk of all-cause mortality
Martens et al. (2018) [7]	ID, IDA and HF with HFrEF, HFmrEF and HFpEF	Serum ferritin < 100 μg/L *or* Serum ferritin 100–300 μg/L + TSAT < 20%	• Lower VO_2max_ • Progression to IDA • Higher risk of HF hospitalization • Higher risk of all-cause mortality
Grote Beverborg et al. (2018) [24]	ID and HF	TSAT ≤ 19.8% + serum iron ≤ 13 μmol/L	• Higher risk of all-cause mortality
Grote Beverborg et al. (2019) [51]	ID and HF	Serum ferritin ≤ 128 µg/L + TSAT < 20%	• Impaired 6MWT • Higher proportion of anemia • Poorer QoL • Higher risk of all-cause mortality • Higher risk of HF hospitalization
		Serum ferritin > 128 µg/L + TSAT < 20%	• Impaired 6MWT • Higher levels of inflammatory markers (CRP, IL-6)
Grammer et al. (2019) [54]	ID/IDA and undergoing coronary angiography	Hb, serum iron, TSAT, sTfR, serum ferritin^#^	• J-shaped associations with cardiovascular and total mortality (marginal for Hb)
		Hepcidin^#^	• Inverse association with mortality
Alcaide-Aldeano et al. (2020) [53]	ID and CHF with HFpEF	Serum ferritin < 100 µg/L *and/or *TSAT < 20% *or* sTfR n/a**	• Worse functional capacity (measured by 6MWT) • Worse QoL (assessed with MLHFQ)
Ambrosy et al. (2020) [55]	Older adults (aged ≥ 65 years) with HF and IDA	**IDA:** Hb < 13 g/dL men *or* < 12 g/dL women + TSAT < 20%	• Higher risk of HF hospitalization • Higher risk of all-cause mortality
Campodonico et al. (2021) [58]	ID and HF	**AID: **serum ferritin < 100 μg/L *or* **FID:** serum ferritin 100–300 μg/L + TSAT < 20%	• Worse prognosis (survival rate) (only for TSAT < 20% *or* serum ferritin 100 – 300 μg/L + TSAT < 20%)
Fitzsimons et al. (2021) [57]	ID and HF (HFpEF, HFrEF) over time (6 months)	**ID**_**Ferritin**_: serum ferritin < 100 μg/L *or *serum ferritin 100–300 μg/L + TSAT < 20% *or* **ID**_**Tsat**_: TSAT < 20%	• Persistent ID_TSAT_ is strongly associated with mortality (only for HFrEF) • ID_Tsat_ is the superior definition of ID

*6MWT* 6-min walking test, *AID* absolute iron deficiency, *AHF* acute heart failure, *CHF* chronic heart failure, *CRP* C-reactive protein, *FID* functional iron deficiency, *Hb* hemoglobin, *HF* heart failure, *HFmrEF* mid-range ejection-fraction, *HFpEF* preserved ejection-fraction, *HFrEF* reduced ejection-fraction, *ID* iron deficiency, *IDA* iron deficiency anemia, IL-6 interleukin 6, *MLHFQ* Minnesota Living with Heart Failure questionnaire, *n/a* not available, *NT-proBNP* N-terminal pro-brain-type natriuretic peptide, *NYHA* New York Heart Association, *QoL* Quality of Life, *sTfR* soluble transferrin receptor, *TSAT* transferrin saturation

^*^ isolated TSAT < 20% had higher NT-proBNP levels and worse QoL compared with isolated serum ferritin < 100 µg/L

^**^ sTfR presented the highest performance as a predictor of functional capacity and QoL

^#^ Iron status (median): iron(μg/dL), 94 men and 81 women; TSAT(%), 27.3 men and 23.4 women; ferritin(ng/mL) 177 men and 99 women; sTfR(mg/L), 1.28 men and 1.23 women; sTfR-F, 0.57 men and 0.66 women; Hb(g/dL), 14.4 men and 13.0 women; hepcidin(ng/mL), 6.6 men and 5.7 women

### Iron deficiency in chronic kidney disease (CKD)

ID is also common among patients with CKD. Several factors, such as reduced iron absorption, blood loss, chronic inflammation, hyperparathyroidism, drugs (e.g., ACE-I, ARB, aspirin) and erythropoietin deficiency/hyporesponsiveness to erythropoietin, might cause the development of ID/ ID anemia in patients with CKD [4, 42]. Based on different study designs and definitions of ID, the prevalence ranges from 24 to 85%, also depending on the CKD stage [4]. In CKD, ID is often associated with a negative clinical impact (Table 3) [4, 65]. However, it should be noted that the diagnosis and treatment of ID without anemia is not well studied in CKD. Irrespective of the presence of ID with or without anemia, the study results provide a correlation between low iron status (measurement with serum iron and/or serum ferritin and/or TSAT) and clinical outcome in patients with non-dialysis (ND)/hemodialysis (HD)-CKD [65–70]. In case of functional ID/anemia, high ferritin levels with a value of > 100–500 or 109–2783 μg/L indicated a higher risk of mortality and cardiovascular hospitalization [66, 67]. In contrast, low TSAT values were significantly associated with a higher mortality risk independent of absolute or functional ID/anemia [68, 71–73]. In an analysis of approx. 2500 ND-CKD patients, a significantly lower physical health-related QoL was found for ID (measured by low TSAT level) after adjustment for Hb levels [45]. Further studies explored TSAT as a marker of iron metabolism by its modifying effects on Hb response in dialysis patients [74]. Therefore, TSAT could be a suitable marker for iron supplementation therapy to achieve clinical improvements [75, 76]. The clinical role of sTfR or sTfR–ferritin index for identifying ID in CKD is limited by the small number of studies and their heterogeneous results [77, 78]. The worse outcome of ID in CKD reflects the major importance of its diagnosis and treatment. This is proven by a study with HD patients treated with iv iron (FCM) and epoetin-alfa, which showed the best survival rates with normal to elevated iron values (Hb > 12 g/dL, TSAT > 25%, serum ferritin > 600 μg/L) [69].

**Table 3:** Iron deficiency-associated outcome in patients with chronic kidney disease (CKD)

Study	Study population	ID definition / iron status	ID / iron status-associated outcome
Kaneko et al. (2003) [75]	ID/IDA and HD-CKD*, treated with rhEPO, iv iron	TSAT level < 20%	• Higher CRP > 5 mg/L level; associated with inflammatory process and EPO resistance → iron marker for iron supplementation therapy
Kalantar-Zadeh et al. (2004) [70]	ID and MHD-CKD, treated with epoetin-alfa, iv iron	Serum iron < 45.3 μg/dL [< 8.1 μmol/L]	• Higher risk of mortality^†^ • Higher risk of hospitalization^†^
Pollak et al. (2009) [69]	IDA and HD-CKD, treated with epoetin-alfa, iv iron	Serum ferritin ≤ 100 μg/L + TSAT ≤ 16%	• Worst long-time survival
		Serum ferritin > 600 μg/L + TSAT > 25%	• Best long-time survival
Koo et al. (2014) [72]	IDA and HD-CKD	TSAT ≤ 20%	• Higher risks of composite cardiovascular and all-cause mortality^§^
Gaweda et al. (2014) [74]	IDA and HD-CKD	TSAT 34%	• Max. Hb response
Hamano et al. (2015) [76]	ID/IDA and HD-CKD*	Serum ferritin > 100 µg/L + TSAT < 20%	• Higher ERIs (ESA resistance index) → iron marker for ESA response
Eisenga et al. (2018) [73]	ID and ND-CKD	TSAT < 10%	• Higher risk of all-cause mortality • Higher risk of cardiovascular mortality • Higher risk for developing anemia
Cho et al. (2019) [66]	ID and ND-CKD with/without diabetic issues	**Abnormal iron balance:** TSAT 0.4–16% *or* 28–99.6%, serum ferritin 0.4–55 μg/L *or* 205–4941 μg/L **FID:** TSAT 0.8–16%, serum ferritin 109–2783 μg/L	• Higher risk of all-cause mortality**
Awan et al. (2019) [67]	IDA and ND-CKD	**AID:** serum ferritin < 100 μg/L + TSAT ≤ 20%	• Higher risk of cardiovascular hospitalization
		**FID:** serum ferritin > 100–500 µg/L + TSAT ≤ 20%	• Higher risk of mortality • Higher risk of cardiovascular hospitalization
Sato et al. (2019) [68]	MHD-CKD* (*evaluated iron profiles*)	TSAT < 20%	• Higher risk of all-cause mortality^#^
Yeh et al. (2019) [71]	HD-CKD with/without PKD (*evaluated iron profiles*)	TSAT ≤ 20%	• Higher risk of mortality^‡^
Mehta et al. (2021) [65]	ID/iron status in CKD	**ID:** serum ferritin 4.85–82.48 µg/L + TSAT 1.28–17.24% **FID:** serum ferritin 157.7–3769.0 µg/L + TSAT 1.28–17.24% **Iron-replete:** serum ferritin 82.49–284.4 µg/L + TSAT 17.25–28.018% **Mixed ID: **serum ferritin 82.49–157.6 µg/L + TSAT 1.28–17.24% **High iron: **serum ferritin 284.4–3769.0 µg/L + TSAT 28.019–87.12% **Nonclassified:** serum ferritin 4.85–82.48 µg/L + TSAT 17.25–87.12 *or* serum ferritin 82.49–284.4 µg/L + TSAT 28.019–87.12% *or* serum ferritin 284.4–3769.0 µg/L + TSAT 17.25–28.018%	• ID independently associated with mortality and heart failure • Mixed ID associated with mortality and ESKD • High iron associated with mortality, heart failure and ESKD • FGF23 mediated the risks of mortality and heart failure conferred by ID
Guedes et al. (2021) (45)	ID and ND-dependent CKD	**AID:** serum ferritin < 50 µg/L + TSAT < 20% **FID:** serum ferritin > 300 µg/L + TSAT < 20%	• Worse physical HRQoL

*AID* absolute iron deficiency, *CKD* chronic kidney disease, *CRP* C-reactive protein, *ESA* erythropoiesis-stimulating agents, *ESKD* end-stage kidney disease, *EPO* erythropoietin, *FGF23* Fibroblast growth factor 23, *FID* functional iron deficiency, *HD* hemodialysis, *Hb* hemoglobin, *HRQoL* health-related quality of life, *ID* iron deficiency, *IDA* iron deficiency anemia, *iv* intravenous, *MHD* maintenance hemodialysis, *ND* non-dialysis, *PKD* autosomal-dominant polycystic kidney disease, *rhEPO* recombinant human erythropoietin, *TSAT* transferrin saturation

^*^ Japanese population

^**^ Outcome was similar between diabetic and non-diabetic subgroups

^#^ Compared with the reference groups with TSAT 20–40% or TSAT > 40%

^†^ Outcomes independent of Hb level, EPO and iron doses

^‡^ In non-PKD group, in comparison to the high TSAT group (≥ 50%)

^§^ Compared with the reference group with TSAT 20–40%

### Iron deficiency in inflammatory bowel disease (IBD)

ID in IBD is mainly caused by chronic inflammation and impaired gastrointestinal iron absorption, bowel resection and bleeding [4]. The prevalence of ID in IBD is not well known and most variable of all chronic diseases in this review. It ranges from 36 – 90% [79] or even from 13–90% [4], depending on the reviewed literature. A more precise prevalence of ID of approx. 45% in IBD, likely due to the selection of uniform ID threshold values from different studies (defined as serum ferritin < 30 μg/L or < 100 μg/L (if CRP is > 5 mg/L or > 10 mg/L) and TSAT < 16% or < 20%), was presented by the review Dignass et al. [5].

A routine monitoring of serum ferritin and TSAT is recommended to detect and treat ID, although most results about iron laboratory parameters derive from expert recommendations or reviews and clinical impact is not well studied [5]. Diagnosis of ID in quiescent IBD can be made by serum ferritin level < 30 µg/L and in active IBD by serum ferritin < 100 µg/L or TSAT < 20%. In case of elevated ferritin level of 100–300 µg/L, TSAT with a cut-off value of < 20% should be added as a complementary parameter to confirm the ID [5]. Clinical data about ID without anemia in gastroenterology are still rare. Most data about ID in IBD were studied in case of anemia [4]. However, one study found a relation between the treatment of ID and an improvement in the QoL of IBD patients [80]. In non-anemic, iron-deficient patients with IBD, the administration of iv iron significantly increased Hb, serum iron, serum ferritin and TSAT levels, which resulted in an improved QoL [80].

### Iron deficiency in cancer

ID is a common concomitant disease in patients with cancer. Iron has an essential role in the cell metabolism and homeostasis, which are mostly affected by cancer and its therapy [6, 11, 81]. The causes of ID in patients with cancer depend on tumor type, localization and extent, but also on its treatment [81]. An ID prevalence of 42.6% defined as TSAT < 20% was identified in oncology patients [6]. ID was more frequent in this trial than ID anemia with a prevalence of 33.0% (Hb ≤ 12 g/dL). Most ID patients showed functional ID (82%; TSAT < 20%, serum ferritin ≥ 30 µg/L), whereas 18% had absolute ID. The highest prevalence was found in pancreatic (63.2%), colorectal (51.9%) and lung cancer (50.7%) [6]. A prospective cohort study found that only one third of cancer patients were tested for iron parameters before the start of an anemia treatment [82]. It appears that the diagnosis of ID is not part of medical routine in oncology [6, 82], although both anemia and ID correlated with tumor stage and disease status and also had a significant impact on QoL [6]. Moreover, a significant correlation between ID prevalence (defined as serum ferritin < 30 µg/L and TSAT < 15%, or functional ID with serum ferritin < 800 µg/L and TSAT < 20%) and tumor response was observed [83]. The use of sTfR/log-ferritin index to detect ID in cancer related anemia compared to non-anemia, identified adverse effect on QoL and overall survival [84]. Overall, oncologic patients with anemia showed worse outcome as those without anemia, related to the progressive stadium of the disease and the fact that anemia can be a limiting factor for cancer treatment [85]. Thus, anemic cancer patients had a higher risk of dose reductions and discontinuation in chemotherapy, poor prognosis and significantly shorter overall survival [86]. Further experimental studies discovered an antitumor effect by macrophage-modulating with iron-nanoparticle, which shows the importance of iron in tumor metabolism [87]. Therefore, the iron status may provide information about medical outcome, making the monitoring and treatment of iron parameters essential. Clinical evidence suggests that treatment of ID with iv iron (with/without ESAs), in contrast to oral iron, improves overall outcomes without increasing the risk of infection or cardiovascular morbidity in oncology patients [86]. Notably, treatment with iv iron (FCM) resulted in sustainable Hb correction in tumor patients with chemotherapy-induced anemia [88]. Compared with oral iron, iv iron reduced blood transfusion requirements and hospital stay [89] and improved QoL in colorectal surgery-treated tumor patients [90].

Finally, ID might be a marker for detecting cancer. Thus, a cohort study showed that men and postmenopausal women with ID defined as TSAT < 15%, had a fivefold increased risk of developing gastrointestinal cancer [91]. Especially, serum iron and TSAT showed an inverse association with the risk of colon cancer [86].

## Conclusion

ID in chronic diseases is a multifactorial and complex co-morbidity influenced by the combination of underlying disease (including the disease stages), treatment modalities and chronic inflammatory states. Different interpretations or definitions of ID in guidelines and studies lead to various diagnostic approaches and variable prevalence data, which could be the reason that ID is not fully recognized as a stand-alone medical condition and often overlooked. Significant differences between the different chronic diseases concerning definition and management of ID could be observed. While in HF many clinical data on prevalence, impact on outcome and treatment of ID especially in correlation with a low TSAT level of < 20% exist, ID without anemia is often still underestimated in other chronic diseases like CKD, IBD and cancer. However, an early diagnosis and subsequent treatment of ID is important, since reduced iron levels were associated with negative clinical outcome, e.g., higher risk of morbidity, mortality and reduced QoL within all indications. A higher hospitalization rate was observed in HF and CKD in correlation with ID. Some studies found a possible relationship between ID and cancer development. There are various diagnostic parameters with different threshold values for ID available. However, diagnosis of ID with average threshold values of TSAT < 20% and serum ferritin < 100–300 µg/L is frequently applied, which is independent of the diagnosis of anemia. As serum ferritin may vary depending on inflammatory status, TSAT with a cut-off value of < 20% may be the first indicator of ID, but also of prognostic value in patients with chronic diseases, particularly in HF.

## Data Availability

Not applicable.
